# Trial of the Pluslife SARS-CoV-2 Nucleic Acid Rapid Test Kit: Prospective Cohort Study

**DOI:** 10.2196/48107

**Published:** 2023-11-14

**Authors:** Dandan Zhu, Jing Huang, Bei Hu, Donglin Cao, Dingqiang Chen, Xinqiang Song, Jialing Chen, Hao Zhou, Aiqun Cen, Tieying Hou

**Affiliations:** 1 Guangdong Center for Clinical Laboratory Guangdong Provincial People’s Hospital (Guangdong Academy of Medical Sciences) Southern Medical University Guangzhou China; 2 Fever Clinic Guangdong Provincial People’s Hospital (Guangdong Academy of Medical Sciences) Southern Medical University Guangzhou China; 3 Department of Emergency Medicine Guangdong Provincial People’s Hospital (Guangdong Academy of Medical Sciences) Southern Medical University Guangzhou China; 4 Department of Laboratory Medicine Guangdong Second Provincial General Hospital Guangzhou China; 5 Microbiome Medicine Center, Department of Laboratory Medicine Zhujiang Hospital Southern Medical University Guangzhou China; 6 Party Committee Office and President Office Guangdong University of Finance and Economics Guangzhou China; 7 Hospital Office Huazhong University of Science and Technology Union Shenzhen Hospital/Shenzhen Nanshan People’s Hospital Shenzhen China

**Keywords:** SARS-CoV-2, COVID-19, RHAM, RNase hybridization-assisted amplification, field trial, diagnosis, screening, rapid test, cohort study, detection, recruitment, infection, Pluslife

## Abstract

**Background:**

In response to the SARS-CoV-2 epidemic, a convenient, rapid, and sensitive diagnostic method for detecting COVID-19 is crucial for patient control and timely treatment.

**Objective:**

This study aimed to validate the detection of SARS-CoV-2 with the Pluslife SARS-CoV-2 rapid test kit developed based on a novel thermostatic amplification technique called RNase hybridization-assisted amplification.

**Methods:**

From November 25 to December 8, 2022, patients with suspected or confirmed COVID-19, close contacts, and health care workers at high risk of exposure were recruited from 3 hospitals and 1 university. Respiratory specimens were collected for testing with the Pluslife SARS-CoV-2 rapid test kit and compared with reverse transcription-quantitative polymerase chain reaction (RT-qPCR) and a commercial antigen assay kit. Samples from 1447 cases were obtained from 3 “ready-to-test” scenarios in which samples were collected on site and tested immediately, and samples from 503 cases were obtained from a “freeze-thaw test” scenario in which samples were collected, frozen, and thawed for testing.

**Results:**

Pluslife SARS-CoV-2 rapid testing of samples from the “ready-to-test” scenario was found to be accurate (overall sensitivity and specificity of 98.3% and 99.3%, respectively) and diagnostically useful (positive and negative likelihood ratios of 145.45 and 0.02, respectively). Pluslife SARS-CoV-2 rapid testing of samples from the “freeze-thaw test” scenario was also found to be accurate (overall sensitivity and specificity of 71.2% and 98.6%, respectively) and diagnostically useful (positive and negative likelihood ratios of 51.01 and 0.67, respectively). Our findings demonstrated that the time efficiency and accuracy of the results in a “ready-to-test” scenario were better. The time required from sample preparation to the seeing the result of the Pluslife SARS-CoV-2 rapid test was 10 to 38 minutes, which was substantially shorter than that of RT-qPCR (at least 90 minutes). In addition, the diagnostic efficacy of the Pluslife SARS-CoV-2 rapid test was better than that of a commercial antigen assay kit.

**Conclusions:**

The developed RNase hybridization-assisted amplification assay provided rapid, sensitive, and convenient detection of SARS-CoV-2 infection and may be useful for enhanced detection of COVID-19 in homes, high-risk industries, and hospitals.

## Introduction

According to the World Health Organization, cases of COVID-19 caused by SARS-CoV-2 were reported in several regions in late 2019 and early 2020 [1]. The rapid spread of SARS-CoV-2 posed a major public health and economic hazard. Currently, the primary test used by most laboratories to diagnose COVID-19 is a nucleic acid amplification test using reverse transcription quantitative polymerase chain reaction (RT-qPCR) [2,3]. Although the World Health Organization declared on May 5, 2023, that the COVID-19 outbreak no longer constituted a “public health emergency of international concern”, this merely demonstrates that the hazard can be effectively controlled with current human capacity—it does not mean that the pandemic is over. The COVID-19 epidemic remains a global health threat, and continued efforts to prevent and control the COVID-19 are still necessary.

For some low-resource primary health care conditions, the application scenarios of traditional nucleic acid testing are limited, and an assay with detection performance comparable to RT-qPCR and less demanding on the testing environment is needed. For high-traffic areas, such as customs and airports, the time required to obtain RT-qPCR results is too long, so a faster and more accurate test is needed to increase the speed of population screening. Therefore, it is essential to develop a rapid, convenient, and accurate method for detecting SARS-CoV-2 with detection performance comparable to that of RT-qPCR for areas where medical resources are scarce and for scenarios with high foot traffic.

Compared with RT-qPCR, thermostatic amplification technology does not require a temperature rise or fall to achieve nucleic acid amplification, which helps to substantially mitigate the cost of the instrument and speed up the reaction process [4-7]. However, while most of the existing traditional thermostatic amplification techniques can solve the problem of instrument cost and fast amplification speed, they suffer from inadequate stability and sensitivity when directly benchmarked against RT-qPCR. Thermostatic amplification technology has been developing for decades, during which a variety of nucleic acid detection methods have emerged [8]. RNase hybridization-assisted amplification (RHAM) is a thermostatic amplification technology developed independently by Guangzhou Pluslife Technology with underlying intellectual property rights. The actual performance of this technology is comparable to that of RT-qPCR, and it is more tolerant and compatible. RHAM can extract, amplify, and detect in 1 step without the opening of the cap after amplification, and it requires a simple professional environment and hardware support. The Pluslife SARS-CoV-2 rapid test kit based on this technology is in lyophilized form, which is easy to transport at room temperature and can achieve extraction-free molecular detection.

In this study, a field trial of the Pluslife SARS-CoV-2 rapid test kit was conducted at 4 centers, including Guangdong Provincial People’s Hospital, Zhujiang Hospital at Southern Medical University, Guangdong Second Provincial General Hospital, and Guangdong University of Finance and Economics. Based on the gold standard RT-qPCR assay, we evaluated the diagnostic performance of the Pluslife SARS-CoV-2 rapid test kit and compared it with a commercial antigen assay kit by Wondfo Biotech for the diagnosis of SARS-CoV-2.

## Methods

### Ethical Considerations

The study protocol was approved by the Ethics Review Committee of Guangdong Provincial People’s Hospital (KY2023-067-02).

### Candidates

**Figure 1:**
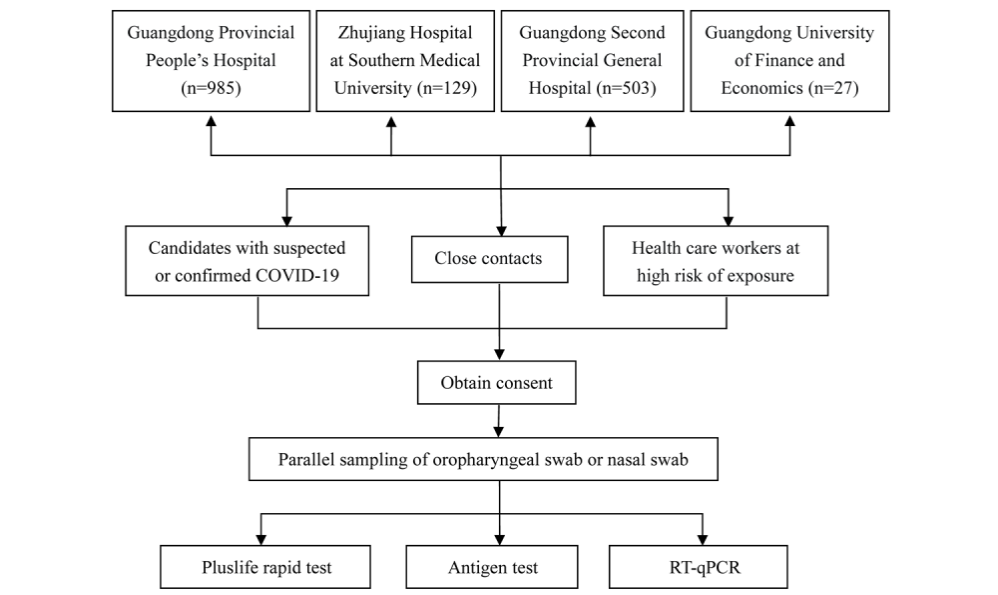
Study flow chart. We recruited candidates with suspected or confirmed COVID-19, close contacts, and health care workers at high risk of exposure at 4 centers. All participants provided written informed consent. Parallel nasal and oropharyngeal swabs were collected and tested with the Pluslife rapid test, antigen test, and RT-qPCR from November 25 to December 8, 2022. RT-qPCR: real-time reverse transcription quantitative polymerase chain reaction.

The study population comprised patients with suspected or confirmed COVID-19, close contacts, and high-risk exposed health care workers from 4 centers, including Guangdong Provincial People’s Hospital, Zhujiang Hospital at Southern Medical University, Guangdong Second Provincial General Hospital, and Guangdong University of Finance and Economics, from November 25 to December 8, 2022. Nasal swabs or oropharyngeal swabs were collected for SARS-CoV-2 testing. The flow chart of the study protocol is shown in Figure 1. The characteristics of the 2028 candidates who received the Pluslife SARS-CoV-2 rapid test are shown in Table 1.

**Table 1:** Characteristics of the 2028 candidates whose samples were tested with the Pluslife SARS-CoV-2 rapid test from November 25 to December 8, 2022.

Characteristic, n			Guangdong Provincial People’s Hospital (n=1333)		Zhujiang Hospital at Southern Medical University (n=147)		Guangdong Second Provincial General Hospital (n=518)		Guangdong University of Finance and Economics (n=30)
**Population**									
	Medical staff (n=1206)	985		121		100		0	
	Teachers (n=11)	0		0		0		11	
	Students (n=8)	0		0		0		8	
	Logistics staff (n=8)	0		0		0		8	
	Fever outpatients (n=213)	205		8		0		0	
	Emergency patients (n=101)	101		0		0		0	
	Confirmed patients with COVID-19 (n=403)	0		0		403		0	
**Collection workers**									
	Grassroots workers (n=1)	0		0		0		1	
	Nurses (n=34)	21		3		10		0	
	Medical self-collection (n=131)	10		121		0		0	
**Testing methods**									
	RT-qPCR^a^ (n=1950)	1291		129		503		27	
	Pluslife rapid test (n=2028)	1333		147		518		30	
	Antigen test (n=283)	283		0		0		0	
**Presence of symptoms**									
	Yes (n=213)	205		8		0		0	
	No (n=1737)	1086		121		503		27	

^a^RT-qPCR: real-time reverse transcription quantitative polymerase chain reaction.

### The RT-qPCR Assay

Viral RNA was extracted from oropharyngeal swabs using the liquid proteinase K magnetic bead method. The BioGerm 2019-nCoV assay (BioGerm) was used to detect SARS-CoV-2 RNA with SARS-CoV-2 RNA-dependent RNA polymerase and nucleocapsid genes according to the product instructions. Briefly, 12 μL of the RT-qPCR mixture, 4 μL of the RT-qPCR enzyme mixture, and 4 μL of the ORFlab/N reaction mixture were combined into a 20 µL master mix for each sample well. The master mix was pipetted into each well of a 96-well optical reaction plate. Then, 5 μL each of specimen nucleic acid, positive control, and negative control were added to the master mix. The tubes were closed securely and centrifuged briefly. Amplification was performed with a fluorescent qPCR instrument for 1 cycle at 50 °C for 10 minutes and 95 °C for 5 minutes, followed by 45 cycles at 95 °C for 10 seconds and 55 °C for 40 seconds. The results of the assay were evaluated with a cycling threshold value, where a value <40 for both target genes was defined as a positive result.

### Pluslife SARS-CoV-2 Card Test Procedure

First, the comprehensive nucleic acid test was preheated. The entire absorbent tip of the disposable sampling swab was gently inserted into one nostril approximately 1.5 to 2 cm deep and rotated 3 times on the inner wall or rubbed 3 times on the posterior pharyngeal wall and both sides of the pharyngeal-palatal arch. For the “ready-to-test” method, the tip of the disposable sampling swab was twisted against the bottom and side of the nucleic acid releaser tube 10 times while pinching the tip of the disposable sampling swab. For the “freeze-thaw test” method, the collected pharyngeal swabs were placed in a 500 μL sample tube of Youkang virus preservation solution, shaken and mixed, and frozen in a –20°C refrigerator for 24 to 240 hours. The sample tubes were thawed and 150 μL of Youkang virus preservation solution was added to 2 mL of nucleic acid release agent, the caps were screwed on, and the tubes were vortexed and mixed for 5 to 10 seconds or mixed by inverting 10 to 15 times. After that, the procedure was the same for both methods. The nucleic acid–releasing agent solution was poured into the SARS-CoV-2 reaction card sample tube between the 2 injection lines. The protruding curved air pocket on the SARS-CoV-2 reaction card sample tube was pressed down, and the card was held and shaken up and down 10 times. The SARS-CoV-2 response card was inserted into the device, and the “Start Test” button was pressed. The red indicator light indicated a positive SARS-CoV-2 nucleic acid result, and the blue indicator light indicated a negative SARS-CoV-2 nucleic acid result. The specific operation process is illustrated in Multimedia Appendix 1.

### Antigen Test Procedure

An antigen detection kit (Wondfo Biotech) was used to detect the SARS-CoV-2 nucleocapsid antigen in respiratory specimens. This assay device contained 2 precoated antibody lines: a “C” (control) line and a “T” (test) line. The control area was precoated with chicken IgY antibody, and the test area was precoated with SARS-CoV-2 antibody. Color particle–coupled anti–SARS-CoV-2 antibody was used to detect SARS-CoV-2 antigen. In the assay, SARS-CoV-2 antigen in the respiratory specimen interacted with the color particle–coupled anti–SARS-CoV-2 monoclonal antibody to form a colored antigen-antibody complex. The complex migrated across the membrane by capillary action up to the test line, where it was captured by the pre-encapsulated anti–SARS-CoV-2 antibody. If SARS-CoV-2 antigen was present in the respiratory specimen, a colored test line was visible in the result window, and the intensity of the stained test line varied with the amount of SARS-CoV-2 antigen in the specimen. Colored particles conjugated to chicken IgY were used as the control line.

### Statistical Analysis

We estimated the minimum sample size using the single group target value method with the following formula:



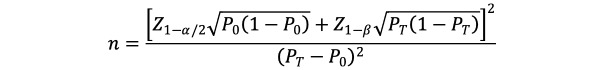



where *n* is the sample size; *Z*_1-α/2_ and *Z*_1-β_ are the significance level and the fractional positions of the standard normal distribution of the degree of certainty, respectively; *P*_0_ is the clinically acceptable standard of the evaluation index; and *P_T_* is the expected value of the evaluation index of the assessment reagent. The positive and negative compliance rates of the assessment reagents and control reagents should reach 80% and 85% clinically, respectively, and the expected positive and negative compliance rates of the assessment reagents and control reagents can reach 90%. The minimum positive and negative sample sizes were estimated to be 137 and 470 cases, respectively, when the significance level α=.05 and the degree of certainty β=.20. Therefore, the minimum total sample size was estimated to be 607 cases. Sensitivity, specificity, positive and negative predictive values, and positive and negative likelihood ratios were calculated using Microsoft Excel 2021 (Microsoft Corp). Sensitivity was calculated as (true positives) / (true positives + false negatives) × 100. Specificity was calculated as (true negatives) / (true negatives + false positives) × 100. Positive predictive value was calculated as (true positives) / (true positives + false positives) × 100. Negative predictive value was calculated as (true negatives) / (true negatives + false negatives) ×100. The positive likelihood ratio was calculated as sensitivity / (1 – specificity). The negative likelihood ratio was calculated as specificity / (1 – sensitivity). *P*<.05 was considered statistically significant. Figures were constructed using GraphPad Prism 8.0 (GraphPad) and R software (R Foundation for Statistical Computing).

## Results

### Comparison of the Pluslife Rapid SARS-CoV-2 Detection Kit With RT-qPCR

The results of the Pluslife rapid SARS-CoV-2 test and RT-qPCR in the 4 centers are shown in Table 2. Of 1950 patients tested using RT-qPCR, a total of 1547 (79.3%) were negative and 403 (20.7%) were positive. Of the 1547 negative patients, 1535 (99.2%, 95% CI 98.8%-99.7%) were also negative when tested with the Pluslife rapid SARS-CoV-2 test. Of the 403 positive patients, 318 (78.9%, 95% CI 76.8%-81%) were also positive when tested with the Pluslife rapid SARS-CoV-2 test. The overall compliance rate was 95% (1853/1950; 95% CI 93.9%-96.2%), with a κ coefficient of 0.84 (95% CI 0.82-0.86). The positive predictive value (PPV) was 96.4% (318/330; 95% CI 95.4%-97.3%), and the negative predictive value (NPV) was 94.8% (1535/1620; 95% CI 93.6%-95.9%). Next, we adopted 2 study protocols, namely, the “ready-to-test” and “freeze-thaw test” protocols, for the field trial scenarios, in which Guangdong Provincial People’s Hospital, Zhujiang Hospital at Southern Medical University, and Guangdong University of Finance and Economics were used in the “ready-to-test” scenarios and Guangdong Second Provincial General Hospital in the “freeze-thaw test” scenario. The results of the Pluslife rapid SARS-CoV-2 test and RT-qPCR are shown in Table 3. RT-qPCR results were negative for 1332 (92%) and positive for 115 (8%) of 1447 patients. Of the 1332 negative patients, 1323 (99.3%, 95% CI 98.9%-99.8%) were also negative when tested with the Pluslife rapid SARS-CoV-2 test. Of the 115 positive patients, 113 (98.3%, 95% CI 98%-100%) were also positive when tested with the Pluslife rapid SARS-CoV-2 test. The overall compliance rate was 99.2% (1436/1447; 95% CI 98.8%-99.7%), with a κ coefficient of 0.95 (95% CI 0.94-0.96). The PPV was 92.3% (113/122; 95% CI 91.3%-94%), and the NPV was 99.8% (1323/1325; 95% CI 99.6%-100%). The results of the “freeze-thaw test” for the Pluslife rapid SARS-CoV-2 test and RT-qPCR are shown in Table 4. A total of 215 (42.7%) of 503 patients received negative RT-qPCR results, and 288 (57.3%) received positive results. Of the 215 negative patients, 212 (98.6%, 95% CI 98%-99.2%) were also negative when tested with the Pluslife rapid SARS-CoV-2 test. Of the 288 patients positive, 205 (71.2%, 95% CI 68.8%-73.5%) were also positive when tested with the Pluslife rapid SARS-CoV-2 test. The overall compliance rate was 82.9% (417/503; 95% CI 81%-84.8%), and the κ coefficient was 0.67 (95% CI 0.64-0.69). The PPV was 98.6% (205/208; 95% CI 97.9%-99.2%), and the NPV was 71.9% (212/295; 95% CI 69.6%-74.2%). These data suggest that the “ready-to-test” scenario was better than the “freeze-thaw test” scenario.

**Table 2:** Performance of the Pluslife SARS-CoV-2 rapid test compared to real-time reverse transcription quantitative polymerase chain reaction (RT-qPCR) for 1950 samples from all trial scenarios.

Pluslife SARS-CoV-2 rapid test results	RT-qPCR results		Performance
	Positive (n=403)	Negative (n=1547)	
Positive (n=330), n	318	12	—^a^
Negative (n=1620), n	85	1535	—
Sensitivity (n=403), n (%, 95% CI)	—	—	318 (78.9, 76.8-81)
Specificity (n=1547), n (%, 95% CI)	—	—	1535 (99.2, 98.8-99.9)
Overall compliance rate (n=1950), n (%, 95% CI)	—	—	1853 (95, 93.9-96.2)
κ (95% CI)	—	—	0.84 (0.82-0.86)
Positive predictive value (n=330), n (%, 95% CI)	—	—	318 (96.4, 95.4-97.3)
Negative predictive value (n=1620), n (%, 95% CI)	—	—	1535 (94.8, 93.6-95.9)
Positive likelihood ratio	—	—	101.73
Negative likelihood ratio	—	—	0.21

^a^Not applicable.

**Table 3:** Performance of the Pluslife SARS-CoV-2 rapid test compared to real-time reverse transcription quantitative polymerase chain reaction (RT-qPCR) for 1447 samples from all “ready-to-test” scenarios.

Pluslife SARS-CoV-2 rapid test results	RT-qPCR results		Performance
	Positive (n=115)	Negative (n=1332)	
Positive (n=122), n	113	9	—^a^
Negative (n=1325), n	2	1323	—
Sensitivity (n=115), n (%, 95% CI)	—	—	113 (98.3, 98-100)
Specificity (n=1332), n (%, 95% CI)	—	—	1323 (99.3, 98.9-99.8)
Overall compliance rate (n=1447), n (%, 95% CI)	—	—	1436 (99.2, 98.8-99.7)
κ (95% CI)	—	—	0.95 (0.94-0.96)
Positive predictive value (n=122), n (%, 95% CI)	—	—	113 (92.3, 91.3-94)
Negative predictive value (n=1325), n (%, 95% CI)	—	—	1323 (99.8, 99.6-100)
Positive likelihood ratio	—	—	145.45
Negative likelihood ratio	—	—	0.02

^a^Not applicable.

**Table 4:** Performance of the Pluslife SARS-CoV-2 rapid test compared to real-time reverse transcription quantitative polymerase chain reaction (RT-qPCR) for 503 samples from all “freeze-thaw test” scenarios.

Pluslife SARS-CoV-2 rapid test results	RT-qPCR results		Performance
	Positive (n=288)	Negative (n=215)	
Positive (n=208), n	205	3	—^a^
Negative (n=295), n	83	212	—
Sensitivity (n=288), n (%, 95% CI)	—	—	205 (71.2, 68.8-73.5)
Specificity (n=215), n (%, 95% CI)	—	—	212 (98.6, 98-99.2)
Overall compliance rate (n=503), n (%, 95% CI)	—	—	417 (82.9, 81-84.8)
κ (95% CI)	—	—	0.67 (0.64-0.69)
Positive predictive value (n=208), n (%, 95% CI)	—	—	205 (98.6, 97.9-99.2)
Negative predictive value (n=295), n (%, 95% CI)	—	—	212 (71.9, 69.6-74.2)
Positive likelihood ratio	—	—	51.01
Negative likelihood ratio	—	—	0.67

^a^Not applicable.

In addition, we compared the testing time of RT-qPCR and the Pluslife rapid SARS-CoV-2 test in the 4 centers, and the results are shown in Table 5. Among participants with negative nucleic acid test results, the mean testing time for RT-qPCR (534.70, SD 466.94 minutes) was substantially longer than that for the Pluslife rapid SARS-CoV-2 test (35.00, SD 0 minutes). Similarly, among participants with positive nucleic acid test results, the testing time for RT-qPCR (882.20, SD 517.97 minutes) was substantially longer than that for the Pluslife rapid SARS-CoV-2 test (16.49, SD 5.51 minutes). Moreover, similar results were obtained when comparing the testing times obtained using the 2 testing methods for the 4 field trial sites (Multimedia Appendix 2).

**Table 5:** Comparison of the testing times of the Pluslife SARS-CoV-2 rapid test and real-time reverse transcription quantitative polymerase chain reaction (RT-qPCR) at the 4 centers.

Testing result		Testing time (min), mean (SD)	*P* value	
**Negative**				<.001
	RT-qPCR	534.70 (466.94)		
	Pluslife SARS-CoV-2 rapid test	35.00 (0)		
**Positive**				<.001
	RT-qPCR	882.20 (517.97)		
	Pluslife SARS-CoV-2 rapid test	16.49 (5.51)		

### Comparison of the Pluslife Rapid SARS-CoV-2 Test and Commercial Antigen Test With the RT-qPCR Nucleic Acid Test

A total of 283 samples that underwent testing using both the commercial antigen test and Pluslife rapid SARS-CoV-2 test were included. RT-qPCR confirmed that there were 93 positive samples and 190 negative samples. The results of the 2 methodological assays compared with the RT-qPCR results are shown in Table 6. The data showed that, compared to the RT-qPCR results, the sensitivity of the commercial antigen test was 88.2% (82/93; 95% CI 86.5%-89.8%) with a specificity of 100%, and the sensitivity of the Pluslife rapid SARS-CoV-2 test was 98.9% (92/93; 95% CI 98.4%-99.4%) with a specificity of 100%.

**Table 6:** Comparison of the results of testing 283 samples using the 2 assays (commercial antigen kit and Pluslife SARS-CoV-2 rapid test kit) with real-time reverse transcription quantitative polymerase chain reaction (RT-qPCR) results.

Assay results		RT-qPCR results		Performance
		Positive (n=93)	Negative (n=190)	
**Commercial antigen test**				
	Positive (n=82), n	82	0	—^a^
	Negative (n=201), n	11	190	—
	Sensitivity (n=93), n (%, 95% CI)	—	—	82 (88.2, 86.5-89.8)
	Specificity (n=190), n (%)	—	—	190 (100)
**Pluslife SARS-CoV-2 rapid test**				
	Positive (n=92), n	92	0	—
	Negative (n=191), n	1	190	—
	Sensitivity (n=93), n (%, 95% CI)	—	—	92 (98.9, 98.4-99.4)
	Specificity (n=190), n (%)	—	—	190 (100)

^a^Not applicable.

### Comparison of the Diagnostic Value of Different Testing Methods and Different Trial Sites

**Figure 2:**
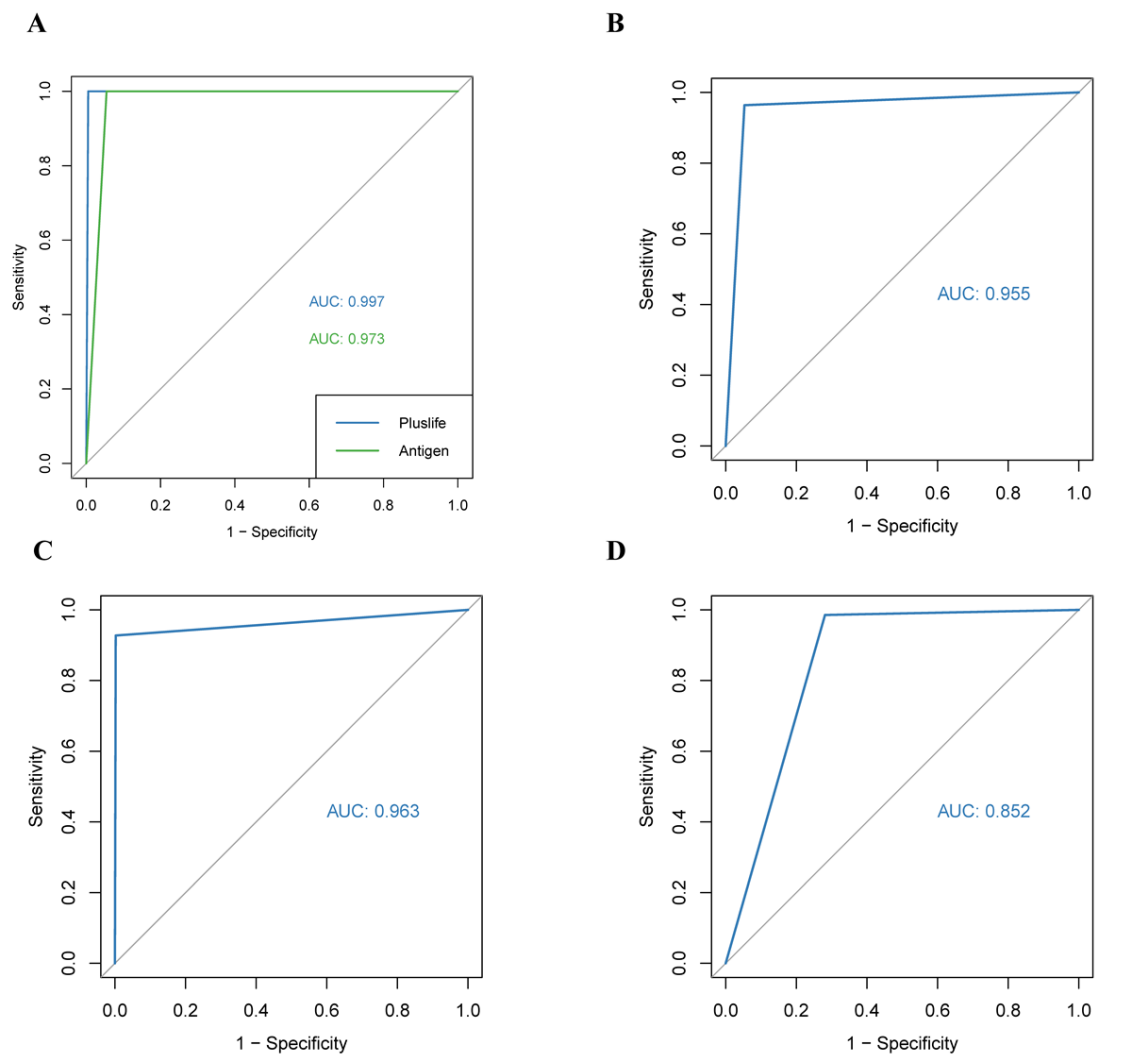
Analysis of the diagnostic value of different assay methodologies and different study protocols. (A) ROC curve analysis comparing the diagnostic value of antigen testing with that of the Pluslife SARS-CoV-2 rapid test. ROC curves predicting the diagnostic value of the Pluslife SARS-CoV-2 rapid test for (B) the 4 centers, (C) the “ready-to-test” study protocol, and (D) the “freeze-thaw test” study protocol. AUC: area under the curve; ROC: receiver operating characteristic.

We performed a receiver operating characteristic (ROC) curve analysis to assess the predictive value of the diagnostic variables. The optimal cutoff values and the area under the receiver operating characteristic curve (AUROC) were first calculated for the commercial antigen test and the Pluslife rapid SARS-CoV-2 test. The AUROCs were 0.973 and 0.997 for the commercial antigen test and the Pluslife rapid SARS-CoV-2 test, respectively (Figure 2A), indicating the higher diagnostic value of the Pluslife rapid SARS-CoV-2 test. After that, the optimal cutoff values and AUROC of the Pluslife rapid SARS-CoV-2 test were calculated for the different study protocols and for each field trial site. The AUROCs were 0.955, 0.963, and 0.852 for the 4 centers combined, the “ready-to-test” scenario, and the “freeze-thaw test” scenario, respectively (Figure 2B-D), indicating that the diagnostic value of testing immediately after field sample collection was higher than that after freezing and thawing samples. The AUROCs of the Pluslife rapid SARS-CoV-2 test for each individual site were 0.959, 1.000, 0.852, and 1.000 for Guangdong Provincial People’s Hospital, Zhujiang Hospital at Southern Medical University, Guangdong Second Provincial General Hospital, and Guangdong University of Finance and Economics, respectively (Multimedia Appendix 3).

## Discussion

### Principal Findings

Molecular testing is currently the standard diagnostic method for confirming SARS-CoV-2 infection [9], and RT-qPCR for SARS-CoV-2 in respiratory specimens is widely used in COVID-19 diagnostic laboratories [2,10-12]. The easy-to-use and mass-producible nature of the Pluslife rapid SARS-CoV-2 test allows for timely nucleic acid test results and reduces the burden on laboratories [10]. Our objective was to evaluate the performance of the Pluslife rapid SARS-CoV-2 test by comparing it with other tests, such as RT-qPCR and antigen tests, through field trials of the Pluslife rapid SARS-CoV-2 test kit at 4 centers.

First, our results from the 4 field trial sites revealed that RT-qPCR showed 95% (1853/1950; 95% CI 93.9%-96.2%) compliance with the results of the Pluslife rapid SARS-CoV-2 test. The specificity, sensitivity, concordance, positive predictive value, negative predictive value, positive likelihood ratio, and negative likelihood ratio of the Pluslife rapid SARS-CoV-2 test were high (Table 2). To test the optimal conditions for the Pluslife rapid SARS-CoV-2 test, we adopted the “ready-to-test” and “freeze-thaw test” protocols in the field trial sites. Guangdong Provincial People’s Hospital, Zhujiang Hospital at Southern Medical University, and Guangdong University of Finance and Economics were used as the test sites for the “ready-to-test” scenario, and Guangdong Second Provincial General Hospital was used as the test site for the “freeze-thaw test” scenario. Our results showed that the RT-qPCR results of the “ready-to-test” and “freeze-thaw test” protocols were 99.2% (1436/1447; 95% CI 98.8%-99.7%) and 82.9% (417/503; 95% CI 81%-84.8%), respectively, and the specificity, sensitivity, concordance, positive predictive value, negative predictive value, positive likelihood ratio, and negative likelihood ratio of the SARS-CoV-2 assay were higher with the Pluslife rapid SARS-CoV-2 test. The above results indicated that freezing and thawing samples after collection and then testing with the Pluslife rapid SARS-CoV-2 test kit is likely to cause false-negative test results, while the diagnostic performance of the test is better when the samples are processed in the field immediately, which may be related to the easy degradation of viral RNA after freezing and thawing. In addition, we validated the diagnostic value of the Pluslife rapid SARS-CoV-2 test by ROC curve analysis for the different study protocols and various field trial sites, and the results also showed that the diagnostic value of the test immediately after field collection was higher than that of the test after freezing and thawing the samples. It is well known that RT-qPCR assay results take a longer time to become available [11]. Our comparison of RT-qPCR and Pluslife rapid SARS-CoV-2 testing times in the 4 centers revealed that the Pluslife rapid SARS-CoV-2 testing time was considerably shorter than the RT-qPCR testing time for both negative and positive results.

Rapid antigen testing is now a valuable alternative to RT-qPCR for the diagnosis of SARS-CoV-2 infection because of its simplicity, speed, low cost, and lack of a need for special equipment or skills [13,14]. However, since viral antigens are expressed when the virus is in active replication, the antigen test is more suitable for the acute infection period, and the accuracy of the COVID-19 antigen test is relatively high for the suspected population within 7 days of the onset of symptoms [15,16]. Therefore, there is a need to develop a test that covers a long period and is fast, accurate, and convenient, which is exactly what the Pluslife rapid SARS-CoV-2 test offers. To compare the diagnostic performance of the Pluslife rapid SARS-CoV-2 test with a commercial antigen test, we compared the 2 assays with RT-qPCR in a field trial scenario at Guangdong Provincial People’s Hospital, and the results showed that the sensitivity of the antigen and Pluslife rapid SARS-CoV-2 tests were 88.2% (82/93; 95% CI 86.5%-89.8%) and 98.9% (92/93; 95% CI 98.4%-99.4%), respectively. Thus, the positive detection rate of the Pluslife rapid SARS-CoV-2 test was increased by 10.8% (10/93; 95% CI 9.2%-12.4%) compared to the commercial antigen test. In addition, we validated the diagnostic value of these 2 tests by ROC curve analysis, and the results showed a higher diagnostic value for SARS-CoV-2 with the Pluslife rapid SARS-CoV-2 test than with the antigen test. Therefore, although the detection time of the Pluslife rapid SARS-CoV-2 test is slightly longer than that of the antigen test, the diagnostic performance of the Pluslife rapid SARS-CoV-2 test for SARS-CoV-2 is superior to that of the antigen test.

However, there are some limitations to our study. First, because most of the samples were collected by health care workers in the 4 centers, it was not possible to compare the effects of the 2 sampling methods, namely, nasal swabs and oropharyngeal swabs, on the performance of the Pluslife rapid SARS-CoV-2 test. Second, because antigen testing was only performed in a subset of patients in the fever clinic of Guangdong Provincial People’s Hospital, the number of samples for which both the Pluslife rapid SARS-CoV-2 test and antigen test were performed was small.

### Conclusion

In conclusion, our multicenter field trial showed that, based on the gold standard RT-qPCR assay, the specificity, sensitivity, concordance, positive predictive value, negative predictive value, positive likelihood ratio, and negative likelihood ratio of the Pluslife rapid SARS-CoV-2 kit were high, and the “ready-to-test” assay conditions were optimal. In addition, the detection time of the Pluslife rapid SARS-CoV-2 test was substantially shorter than that of RT-qPCR, which helps to reduce laboratory pressure and time costs with better performance in the detection of SARS-CoV-2. Furthermore, when compared with the antigen test, we found that the diagnostic performance of the Pluslife rapid SARS-CoV-2 test was better for SARS-CoV-2 with similar detection times. Therefore, under “ready-to-test” assay conditions, the Pluslife rapid SARS-CoV-2 test can be used as a fast, convenient, and accurate method for detecting SARS-CoV-2 with a detection performance comparable to that of RT-qPCR, offering significant application value for grassroots level facilities with poor medical conditions, special application scenarios (eg, traffic checkpoints), and easy home testing, thus reducing pressure on medical institutions.

## Appendix

Multimedia Appendix 1 Pluslife SARS-CoV-2 card test procedure.

Multimedia Appendix 2 Comparison of the testing times of the Pluslife SARS-CoV-2 rapid test and real-time reverse transcription quantitative polymerase chain reaction (RT-qPCR) at Guangdong Provincial People's Hospital, Zhujiang Hospital at Southern Medical University, Guangdong Second Provincial General Hospital, and Guangdong University of Finance and Economics.

Multimedia Appendix 3 Receiver operating characteristic curves predicting the diagnostic value of the Pluslife SARS-CoV-2 rapid test at (A) Guangdong Provincial People’s Hospital, (B) Zhujiang Hospital at Southern Medical University, (C) Guangdong Second Provincial General Hospital, and (D) Guangdong University of Finance and Economics. AUC: area under the curve.

## Abbreviations

AUROC: area under the receiver operating characteristic curve
RHAM: RNase hybridization-assisted amplification
ROC: receiver operating characteristic
RT-qPCR: real-time reverse transcription quantitative polymerase chain reaction

## Data Availability

The data sets generated and analyzed during this study are not publicly available due to participant privacy concerns but are available from the corresponding author on reasonable request.
