# Supplementary material for: Trial of the Pluslife SARS-CoV-2 Nucleic Acid Rapid Test Kit: Prospective Cohort Study
Source: JMIR Public Health Surveill. 2023 Nov 14;9:e48107. doi: 10.2196/48107 (PMC10650960; doi:10.2196/48107)
Supplement: Multimedia Appendix 1 [file publichealth_v9i1e48107_app1.pdf]

01 Instrument Preparing

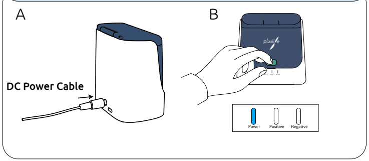

A. Connect DC power cable to power supply.
B. Press the button to start warm-up (about 2 mins). When the power light turns blue, warm-up is complete.

02 Sampling

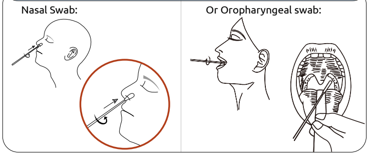

Nasal Swab: have the test subject wipe away excess mucus, if present. Rotate swab 3 times in each nostril, respectively.
Or Oropharyngeal swab: the person being tested needs to tilt the head slightly and open the mouth wide. Rub swab over both tonsillar pillars and posterior oropharynx at least 3 times.

03 Sample Processing

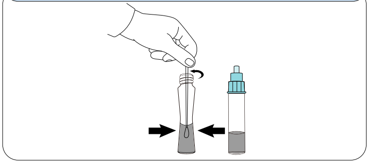

Rotate the swab in the Nucleic Acid Releasing Agent 01 Vial 10 times while pinching the swab by the tip. Discard the swab and screw on the cap.

04 Transferring to Reaction Card

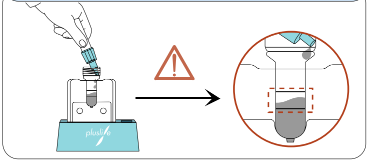

Pinch the vial to slowly pour the releasing agent solution on the tube inside wall of Reaction Card. MAKE SURE the liquid is between the two level lines.

05 Screw on the Cap Correctly

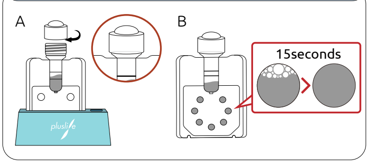

A. Screw the tube cap of the Card until it is tight . If it is not airtight, the test could fail or give an invalid result.
B. Allow the card stand still for 15 seconds.

06 Moving Liquid into Reaction Chamber

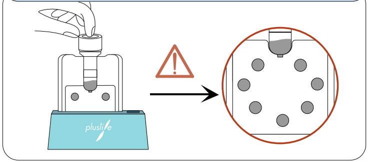

Firmly press the air button on top of the Card cap. Ennsure that ALL chambers are completely filled with liquid.

07 Mixing

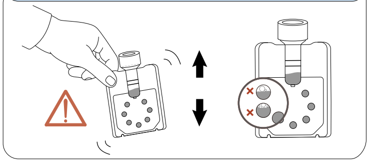

Quickly shake the Card up and down 10 times within 5 seconds. Discard the card if the bubble volume occupies more than 1/3 of the chamber.

08 Amplification

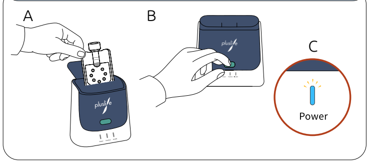

A. Insert the reaction Card into Mini Dock and close the top cover.
B. Press the button to start amplification.
C. The power light will flash blue during the amplification process. Read the results after 15 to 35 minutes.

09 Test Results

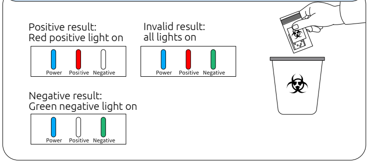

The test results are as above. After the test, dispose of the reaction card in the waste bag and seal the bag ASAP.
If move on to next test, press the power button to eliminate the last test result (the power indicator is steady on), then back to STEP1. If not, press the button for over 3 seconds to turn it off.
