# Supplementary material for: Trial of the Pluslife SARS-CoV-2 Nucleic Acid Rapid Test Kit: Prospective Cohort Study
Source: JMIR Public Health Surveill. 2023 Nov 14;9:e48107. doi: 10.2196/48107 (PMC10650960; doi:10.2196/48107)
Supplement: Multimedia Appendix 2 [file publichealth_v9i1e48107_app2.docx]

**Multimedia Appendix 2.**

**Table S1**. Comparison of the testing times of the Pluslife SARS-CoV-2 rapid test and real-time reverse transcription quantitative polymerase chain reaction (RT-qPCR) at Guangdong Provincial People’s Hospital.

| Testing result | | Testing time (min), mean (SD) | *P* value |
| --- | --- | --- | --- |
| **Negative** | | | P<0.0001 |
|  | RT-qPCR | 296.10 (158.44) |  |
|  | Pluslife SARS-CoV-2 rapid test | 35.00 (0) |  |
| **Positive** | | | P<0.0001 |
|  | RT-qPCR | 294.40 (235.70) |  |
|  | Pluslife SARS-CoV-2 rapid test | 18.22 (6.85) |  |

**Table S2**. Comparison of the testing times of the Pluslife SARS-CoV-2 rapid test and real-time reverse transcription quantitative polymerase chain reaction (RT-qPCR) at Zhujiang Hospital at Southern Medical University.

| Testing result | | Testing time (min), mean (SD) | *P* value |
| --- | --- | --- | --- |
| **Negative** | | | P<0.0001 |
|  | RT-qPCR | 318.80 (164.96) |  |
|  | Pluslife SARS-CoV-2 rapid test | 35.00 (0) |  |
| **Positive** | | | P=0.02 |
|  | RT-qPCR | 682.5 (410.45) |  |
|  | Pluslife SARS-CoV-2 rapid test | 22.75 (8.38) |  |

**Table S3**. Comparison of the testing times of the Pluslife SARS-CoV-2 rapid test and real-time reverse transcription quantitative polymerase chain reaction (RT-qPCR) at Guangdong Second Provincial General Hospital.

| Testing result | | Testing time (min), mean (SD) | *P* value |
| --- | --- | --- | --- |
| **Negative** | | | P<0.0001 |
|  | RT-qPCR | 1033.00 (518.80) |  |
|  | Pluslife SARS-CoV-2 rapid test | 35.00 (0) |  |
| **Positive** | | | P<0.0001 |
|  | RT-qPCR | 1030.00 (472.16) |  |
|  | Pluslife SARS-CoV-2 rapid test | 15.40 (4.10) |  |

**Table S4**. Comparison of the testing times of the Pluslife SARS-CoV-2 rapid test and real-time reverse transcription quantitative polymerase chain reaction (RT-qPCR) at Guangdong University of Finance and Economics.

| Testing result | | Testing time (min), mean (SD) | *P* value |
| --- | --- | --- | --- |
| **Negative** | | | P<0.0001 |
|  | RT-qPCR | 463.50 (199.77) |  |
|  | Pluslife SARS-CoV-2 rapid test | 35.00 (0) |  |
| **Positive** | | | P<0.001 |
|  | RT-qPCR | 429.70 (235.23) |  |
|  | Pluslife SARS-CoV-2 rapid test | 19.50 (6.91) |  |
