# Supplementary figures and images for: Trial of the Pluslife SARS-CoV-2 Nucleic Acid Rapid Test Kit: Prospective Cohort Study
Source: JMIR Public Health Surveill. 2023 Nov 14;9:e48107. doi: 10.2196/48107 (PMC10650960; doi:10.2196/48107)

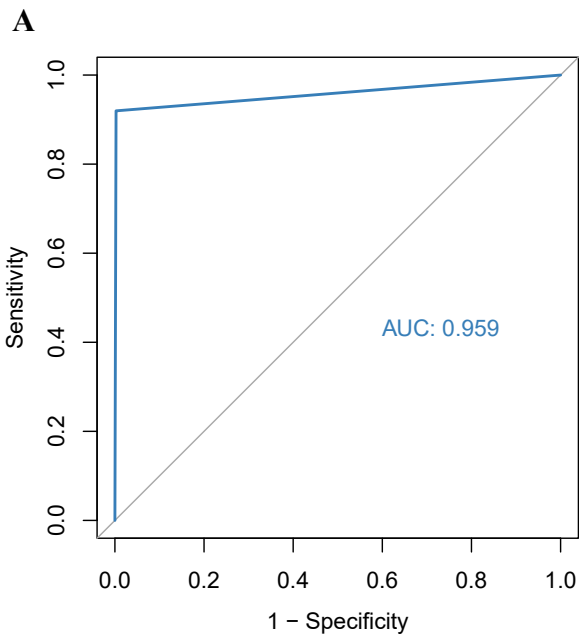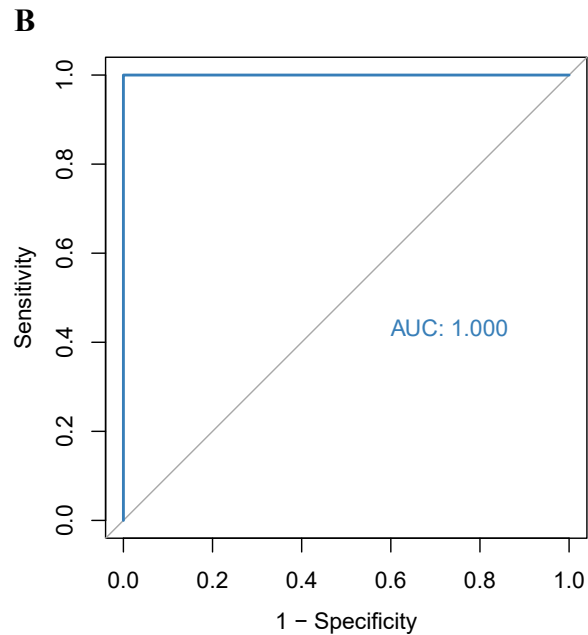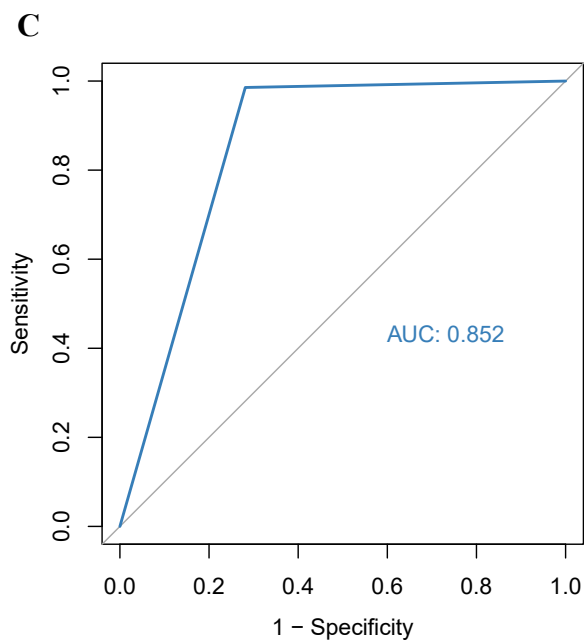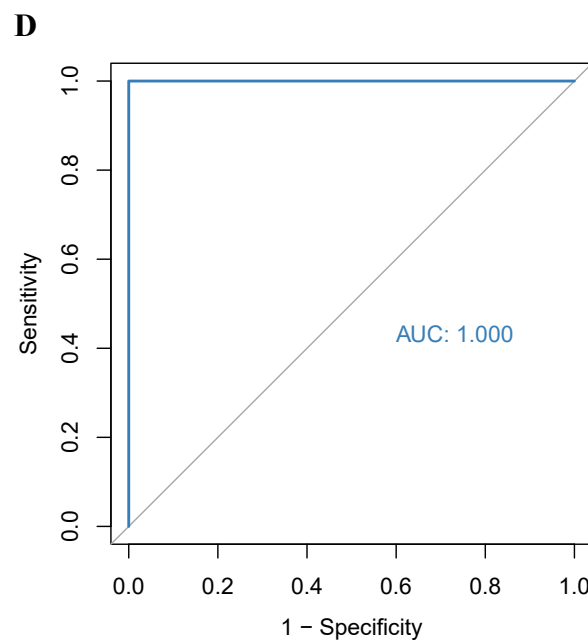

Supplement: Multimedia Appendix 3 [file publichealth_v9i1e48107_app3.pdf]
